# Targeting EphA2 in cancer

**DOI:** 10.1186/s13045-020-00944-9

**Published:** 2020-08-18

**Authors:** Ta Xiao, Yuhang Xiao, Wenxiang Wang, Yan Yan Tang, Zhiqiang Xiao, Min Su

**Affiliations:** 1 Institute of Dermatology, Chinese Academy of Medical Sciences & Peking Union Medical College, Nanjing, Jiangsu 210042 China; 2 Research Center of Carcinogenesis and Targeted Therapy, Xiangya Hospital, Central South University, Changsha, Hunan 410008 China; 3 Thoracic Surgery Department 2, Hunan Cancer Hospital and The Affiliated Cancer Hospital of Xiangya School of Medicine, Central South University, Changsha, Hunan 410013 China; 4 Hunan Key Laboratory of Translational Radiation Oncology, Hunan Cancer Hospital and The Affiliated Cancer Hospital of Xiangya School of Medicine, Central South University, Changsha, 410013 China

**Keywords:** EphA2 receptor, Ephrin A1, Cancer, Therapy, Target

## Abstract

Eph receptors and the corresponding Eph receptor-interacting (ephrin) ligands jointly constitute a critical cell signaling network that has multiple functions. The tyrosine kinase EphA2, which belongs to the family of Eph receptors, is highly produced in tumor tissues, while found at relatively low levels in most normal adult tissues, indicating its potential application in cancer treatment. After 30 years of investigation, a large amount of data regarding EphA2 functions have been compiled. Meanwhile, several compounds targeting EphA2 have been evaluated and tested in clinical studies, albeit with limited clinical success. The present review briefly describes the contribution of EphA2-ephrin A1 signaling axis to carcinogenesis. In addition, the roles of EphA2 in resistance to molecular-targeted agents were examined. In particular, we focused on EphA2’s potential as a target for cancer treatment to provide insights into the application of EphA2 targeting in anticancer strategies. Overall, EphA2 represents a potential target for treating malignant tumors.

## Introduction

Ephrin receptors (Eph) represent the most important class of receptor tyrosine kinases (RTKs) [1]. EphA1, the firstly described Eph receptor, was identified in liver cancer cells while screening for RTKs in 1987 [2]. Nowadays, there are 14 Eph receptors and 8 related ligands (ephrins) [3]. Eph receptor signaling contributes to multiple biological events, mostly causing cell-cell repulsion or adhesion. Therefore, Eph receptors and the corresponding ligands have essential functions in tissue patterning, neuronal targeting, and blood vessel development in the embryo [4, 5]. Meanwhile, Eph proteins are found in high levels in multiple malignancies, with such overexpression significantly contributing to carcinogenesis [6].

Eph receptors are single transmembrane proteins with extra- (N-terminal) and intracellular domains with ligand-binding and intrinsic enzymatic activities, respectively [7, 8]. Eph receptors are grouped into A and B categories according to their extracellular domains, which determine the binding affinity for ligands (Eph receptor-interacting proteins or ephrins) [9, 10]. Nine EphA and five EphB receptors are found in humans [11]. The ligands for Eph receptors, ephrins, are anchored to the cell membrane; they also comprise two subcategories, including ephrin A (ephrin A1-5) and ephrin B (ephrin B1-3) [12, 13].

**Fig. 1:**
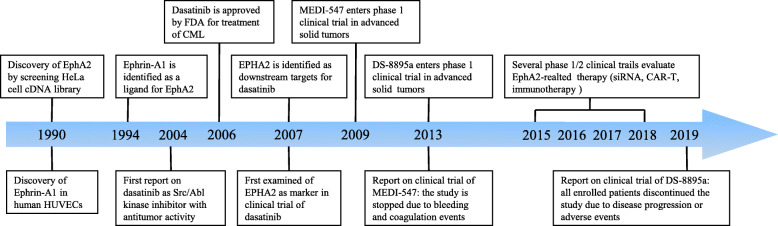
Historical development and breakthroughs in targeting EphA2 in cancer

Some Eph receptors, especially EphA2, attract increasing attention because of demonstrated or hypothesized contributions to modulatory processes controlling carcinogenesis and tumor progression (Fig. 1). The present manuscript reviewed the clinical associations and biological and cellular consequences of EphA2 overexpression in cancer. Potential opportunities for therapeutic intervention based on EphA2 targeting are particularly discussed.

## EphA2-ephrin A1 signaling

The EphA2 receptor is a 130-kDa transmembrane glycoprotein with 976 amino acids [14]. The EphA2 gene in humans is found on chromosome 1p36. Its initial detection occurred in 1990 while screening a HeLa cell cDNA library comprising degenerate oligonucleotides engineered to interact with highly conserved domains of tyrosine kinases [12]. EphA2 was originally termed epithelial cell kinase (eck) since it was detected in most epithelial cells.

EphA2 interacts with any of the eight different ephrin A-family ligands, with overt preference to ephrin A1 [13, 15]. Ephrin A1 represents a GPI-anchored protein containing 205 amino acids (apparent molecular weight, 22 kDa) [16]. The human ephrin A1 gene is located on 1q21-q22. This TNF-α early-inducible gene product was firstly described in human umbilical vein endothelial cells (HUVECs) three decades ago [17], and shown to bind EphA in 1994 [18]. Ephrin A1’s expression pattern in cancer seems to differ from that of EphA2, with attenuation in a variety of aggressive tumors, particularly those overexpressing EphA2 [16].

**Fig. 2:**
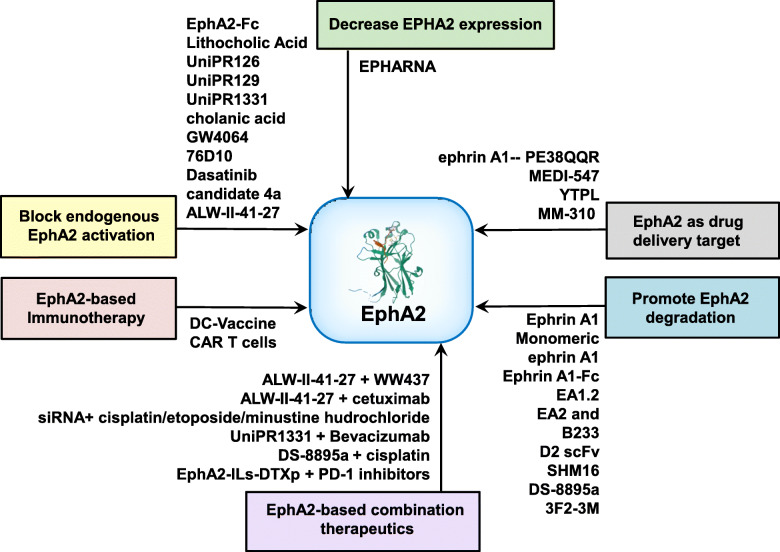
Expression and biological pathways linked with EphA2. The interaction of cell-membrane-bound EphA2 with ephrin A1 induces forward or reverse signals in the corresponding cells. Under normal conditions, cell–cell contacts allow EphA2 to interact with ephrin A1, which induces EphA2 phosphorylation and activates its downstream signaling. Tyrosine phosphorylation of EphA2 promotes the generation of a complex with c-Cbl, subsequently induces EphA2 degradation. This leads to suppression of ECM attachment, cell proliferation, cell migration, and angiogenesis. In the malignant state, loss of cell–cell contacts induces receptor-ligand interaction and degradation of EphA2. In addition, tyrosine phosphorylation of EphA2 could be rapidly reversed by the phosphatase LMW-PTP, further leading to the overexpression and accumulation of unphosphorylated form of EphA2. This leads to promotion of ECM attachment, cell proliferation, cell migration, and angiogenesis

Under normal conditions, EphA2 interacts with ephrin A1 on the neighboring cell and induce diverse signaling networks following cell-to-cell contact. As membrane proteins, ephrins are engaged in both forward (termed ephrin:EphA2 forward) and reverse (called EphA2:ephrin reverse) signaling from ephrin ligands to EphA2 and vice versa; this is also known as ephrin-EphA2 bidirectional signaling [19, 20]. Forward signaling is often cell repulsive and promotes EphA2 oligomerization and phosphorylation, therefore enhancing kinase activity. The immediate biological consequences of EphA2 phosphorylation include decreased cell–extracellular matrix (ECM) attachment. Ephrin A1-associated EphA2 induction inhibits focal adhesion kinase (FAK), extracellular regulated protein kinases (ERK), and Akt phosphorylation to regulate motility, viability, and proliferation in multiple malignant cell lines [7, 21], whereas reverse signaling is more likely to be adhesive and is generally considered as kinase-independent, due to lacking enzyme activity in ephrin A1. However, the reverse signaling by ephrin A1 is largely poorly understood. In addition, EphA2 possesses ligand-independent kinase activity in cultured cancer cells, which might partially explain its malignant effects in the non-phosphorylated state [22, 23]. Actually, EphA2-ephrin A1 interaction or EphA2 ligand-independent kinase activity likely functions through multiple factors acting jointly, e.g., cell type and the microenvironment. Altogether, the EphA2-ephrin A1 signaling regulates multiple cellular processes (proliferation, survival, migration, morphology, cell-to-cell repulsion, and adhesion) in embryonic development, angiogenesis, and tumorigenesis [11] (Fig. 2).

## EphA2 in cancer

Different from the majority of Eph kinases that are mostly synthesized during the developmental process, EphA2 is mainly restricted to proliferating epithelial cells in adults [12]. EphA2 expression in the adult occurs in normal tissues only when they have highly proliferating epithelial cells [1], where its importance and function are not well understood. However, an accumulating body of evidence suggests human EphA2 is abundantly expressed in diverse cancers such as prostate [24], lung [25], esophageal [26], colorectal [27], cervical [28], ovarian [29], and breast [30] and skin cancers [31]. EphA2 is upregulated at the gene and protein levels in human tumor tissue specimens and established cancer cell lines [9, 16]. In particular, most elevated EphA2 expression is consistently detected in cells with highest malignancy [16]. In addition, EphA2 expression has associations with poor prognosis, elevated metastatic potential, and reduced survival of tumor patients [32, 33]. Moreover, EphA2 is not simply a biomarker of malignant character, but also an active participant in malignant progression [26, 28]. Consequently, EphA2’s expression patterns and functional relevance in malignancies make this protein an attractive therapeutic target in cancer.

There is considerable interest in the mechanisms that govern EphA2 expression and in understanding how these mechanisms are subverted in cancer. Emerging evidence links high EphA2 protein amounts with EphA2 regulation at the mRNA level as well as protein stability, although the precise mechanisms governing EphA2 upregulation in cancer remain largely undefined [16, 34].

EphA2 mRNA is tightly regulated. To date, a few somatic mutations of EphA2 have been reported [35–37]. In addition, EphA2 amplification detected in only a low percentage of cases (1 in 33 pancreatic cancer samples) [38]. The EphA2 promoter comprises DNA damage-responsive p53-binding sites, and this receptor is upregulated by ultravioletray (UV) treatment [39]. EphA2 is overexpressed in Ras-transformed cells and transgenic mice overexpressing Ras, suggesting EphA2 as a direct transcriptional target of rat sarcoma (Ras)–(rapidly accelerated fibrosarcoma) Raf–ERK signaling [22, 40]. EphA2 gene expression is also reduced by multiple stimuli such as signaling by the c-Myc and estrogen receptor [41]. These observations are intriguing given that EphA2 consistently shows highest expression in breast tumor cells with most pronounced aggressiveness and no expression of estrogen receptor (ER)-α [41, 42]. Thus, it is tempting to speculate that EphA2 overexpression in breast cancer might be linked to the loss of hormone dependence that frequently arises in advanced stages of the disease.

Decreased ligand-mediated receptor internalization and degradation, consequently enhancing protein stability, might help increase EphA2 amounts in malignant cells. An interesting consequence of EphA2 stimulation (by ligand or antibody) is EphA2 phosphorylation, internalization, and degradation [43–45]. After ligand-dependent induction, EphA2 aggregation occurs at the cell surface, followed by tyrosine phosphorylation, promoting the generation of a complex with c-Cbl, which is internalized into early endosomes for subsequent EphA2 degradation [46]. Studies have shown that c-Cbl overexpression decreases the levels of the EphA2 protein, likely by enhancing protein degradation. Tyrosine phosphorylation of EphA2 could also be rapidly reversed by low-molecular-weight protein phosphatase (LMW-PTP), a phosphatase binding to and dephosphorylating EphA2 [47]. Increased LMW-PTP expression functions to reduce EphA2 phosphotyrosine content, contributing to elevated EphA2 levels in cancer cells. Despite EphA2 overexpression in cancer, phosphorylated EphA2 is found in lower amounts in cancer cells in comparison with non-transformed epithelial cells [42]. Unlike many other receptor tyrosine kinases, the enzymatic activity of EphA2 does not depend on ligand interaction or receptor autophosphorylation [34, 39, 42]. It is considered that deficient cell-to-cell contact (commonly found in malignant cells) and insufficient levels of ephrin A1 on cancer cells reduce EphA2 phosphorylation [48].

## Targeting EphA2 in cancer

**Fig. 3:**
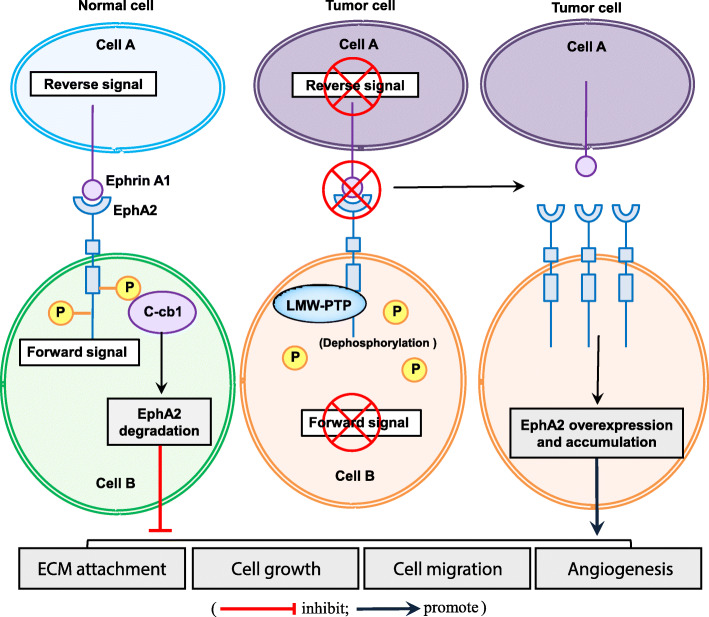
Targeting EphA2 in cancer. EphA2’s expression patterns and functional relevance in malignancies make this protein an attractive therapeutic target in cancer. Accordingly, EphA2 overexpression has been targeted with several approaches such as decrease EPHA2 expression, promote EphA2 degradation, block endogenous EphA2 activation, EphA2 as drug delivery target, EphA2-based immunotherapy, and EphA2-based combination therapeutics

Overexpression and aggressive features of EphA2 in tumor cells and relatively low expression in most normal adult tissues make this protein a potential therapeutic target in cancer. The EphA2/ephrin A1 system could be targeted for cancer treatment at least via two mechanisms. First, EphA2’s oncogenic features could be inhibited, e.g., decreasing EphA2 expression, promoting EphA2 degradation, and blocking endogenous EphA2 activation. Alternatively, the EphA2 receptor could be employed to deliver therapeutics (exogenous drugs or endogenous immune cells) to cancer cells and associated vessels. Therapies targeting EphA2 in cancer are shown in Table 1 and Fig. 3.

**Table 1:** Summary of EphA2 targeted therapies against cancer

Mechanism	Method or compound	Cancer type	Exact effects on EphA2	Effects in vitro	Effects in vivo	Ref.
**Decrease EphA2 expression**						
	EPHARNA	Ovarian cancer	Decrease in vivo EphA2 expression	–	Inhibit tumor growth	[49]
**Promote EphA2 degradation**						
	**Soluble ephrin A1 and ephrin A1-Fc**					
	Ephrin A1	Glioblastoma multiforme	Induce EphA2 internalization and downregulation	Inhibit cell migration		[50]
	Monomeric ephrin A1	Breast cancer	Induce EphA2 phosphorylation and degradation	–	–	[51]
	Ephrin A1-Fc	Pancreatic cancer	Induce EphA2 degradation	Inhibit cell motility and invasion	–	[52]
	Ephrin A1-Fc	Gastric cancer	Induce EphA2 phosphorylation and degradation	Inhibit cell growth	–	[53]
	**EphA2 monoclonal antibody**					
	EA1.2	Breast cancer	Induce EphA2 phosphorylation and degradation	Inhibit cell growth, disrupt angiogenesis	–	[45]
	EA2 and B233	Breast cancer	Induce EphA2 phosphorylation and degradation	inhibit tumor growth in vivo	Inhibit tumor growth	[54]
	D2 scFv	Lymphoma	Prevent EphA2-ephrin interaction	Inhibit cell proliferation, induce apoptosis	–	[55]
	SHM16	Melanoma	Antibody internalization	Inhibit cell migration and invasion	–	[43]
	DS-8895a	Breast cancer and gastric cancer	Inhibit EphA2 phosphorylation	–	Inhibit tumor growth	[56]
	DS-8895a	Breast cancer and gastric cancer	–	–	Inhibit tumor growth	[57]
	3F2-3M	Breast, ovarian, non-small cell lung cancer	Induce EphA2 phosphorylation	Kill tumor cells in vitro	Inhibit tumor growth	[44]
**Block endogenous EphA2 activation**						
	**Inhibit Eph-ephrin interactions**					
	EphA2-Fc	Pancreatic	Inhibit EphA2 phosphorylation	Inhibit angiogenesis	Inhibit tumor growth	[58]
	Lithocholic acid	Prostate and colon cancer	Inhibit EphA2 phosphorylation	Inhibit cell rounding, retraction	–	[59]
	UniPR126	Prostate cancer	Inhibit EphA2 phosphorylation	Inhibit cell rounding, retraction	–	[60]
	UniPR126	Prostate cancer	Inhibit EphA2 phosphorylation	–	–	[61]
	UniPR129	Prostate cancer	Inhibit EphA2 phosphorylation and block kinase domain enzymatic activity	Inhibit cell rounding, disrupt angiogenesis		[62]
	UniPR1331	Prostate cancer	block EphA2 phosphorylation and activation	Disrupt angiogenesis	–	[63]
	Cholanic acid	Prostate cancer	Inhibit EphA2 phosphorylation	Inhibit cell retraction	–	[64]
	GW4064	Prostate cancer	Inhibit EphA2 phosphorylation	–	–	[65]
	76D10	Prostate cancer	Inhibit EphA2 phosphorylation	Inhibit cell retraction	–	[66]
	**Inhibit kinase activity of EphA2**					
	Dasatinib	Melanoma	Inhibit EphA2 phosphorylation and kinase activity	Inhibit cell migration and invasion	–	[67]
	Dasatinib	Pancreatic cancer	Inhibit EphA2 phosphorylation and kinase activity	Inhibit cell growth	–	[68]
	Candidate 4a	Glioblastoma	–	Inhibit cell survival	–	[69]
	ALW-II-41-27	Non-small cell lung cancer	Inhibit EphA2 phosphorylation	Inhibit cell survival	Inhibit tumor growth	[70]
	ALW-II-41-27	Lung cancer	–	Inhibit cell survival, proliferation, migration, increased apoptosis	Inhibit tumor growth	[25]
**EphA2 as drug delivery target**						
	**Peptide/antibody-drug conjugates**					
	Ephrin A1–PE38QQR	Glioblastoma multiforme	Decrease EphA2 expression	Inhibit cell survival	–	[71]
	MEDI-547	Prostate cancer	Induce EphA2 phosphorylation and degradation	Inhibit cell survival	Inhibit tumor growth	[72]
	MEDI-547	Endometrial cancer	Induce EphA2 internalization and degradation	Inhibit cell survival, induce apoptosis	Inhibit tumor growth	[73]
	MEDI-547	Ovarian cancer	Induce EphA2 degradation	Inhibit cell survival and proliferation, induce apoptosis	Inhibit tumor growth	[74]
	**Antibody-directed nanotherapeutics**					
	YTPL	Melanoma	–	Inhibit cell survival	–	[75]
	MM-310	Breast, prostate, gastric, and esophageal cancer	–	–	Inhibit tumor growth	[76]
**EphA2-based immunotherapy**						
	DC-vaccine	Colon cancer (murine)	–	–	Inhibit tumor growth	[77]
	DC-vaccine	Colon cancer (murine) and melanoma (human)	–	–	Inhibit tumor growth	[78]
	CAR T cells	Glioblastoma	Decrease EphA2 expression	–	Inhibit tumor growth	[79]
	CAR T cells	Glioma	–	–	Inhibit tumor growth	[80]
	CAR T cells	Lung cancer	–	Inhibit cell survival	Inhibit tumor growth	[81]
	CAR T cells	Esophageal squamous cell carcinoma	–	Inhibit cell survival	–	[82]

### Inhibiting EphA2 expression

Given the positive association of EphA2 overexpression with aggressive clinical and pathological features in human cancers, investigators have examined the potential of downregulating EphA2 in preclinical models. Short interfering RNAs (siRNAs) for gene knockdown constitute a great tool for protein function assessment, gene discovery, and drug development [83, 84], and have been applied to silence EphA2 in human cancer cells. For example, in pancreatic adenocarcinoma-derived cells, sequence-specific siRNA targeting EphA2 suppresses EphA2 expression, retarding tumor growth in a nude mouse xenograft model [85]. In addition, treatment with EphA2-specific siRNA significantly reduces malignancy in glioma [86], non-small cell lung cancer (NSCLC) [70], and breast cancer cells [87]. However, despite the great success in in vitro knockdown, in vivo siRNA delivery is challenging [88, 89]. As a result, efficient and biocompatible delivery systems for systemic siRNA administration have been evaluated. For instance, EPHARNA, the 1,2-dioleoyl-sn-glycero-3-phosphatidylcholine (DOPC) nanoliposomal EphA2-targeted therapeutic, has been developed [49]. In the nude mouse model administered ovarian tumors intraperitoneally, EPHARNA was shown to be taken up by the tumor, reducing EphA2 levels in the animals 48 h following single treatment [49]. This finding indicates that treatment with EPHARNA reduces tumor growth in the ovarian cancer mouse xenograft model. In addition, both signal dosing and multi-dosing of EPHARNA have an excellent safety profile in many mammalian species, including non-human primates [90].

### Promoting EphA2 degradation

Artificial ligands or antibodies interacting with EphA2 could suppress signaling by promoting internalization and degradation.

#### Soluble ephrin A1 and ephrin A1-Fc

Plasma membrane–bound ephrins and soluble ephrins with artificial clustering/dimerization associated with antibodies targeting COOH-terminal epitope tags or fusion to immunoglobulin g (IgG) Fc potently promote EphA2 phosphorylation and degradation [13].

Ephrin A1 has been demonstrated to be present at low levels and to possess tumor suppressing properties dependent on cell-to-cell contact in a variety of tumors [6, 30, 91]. Transfection with full-length human ephrin A1 into glioblastoma multiforme (GBM) cells exhibits a dramatic suppression of EphA2 and inhibits multiple malignant features, including impaired anchorage-independent growth, proliferation, and migration [50]. Of great interest, ephrin A1 was shown to be released as a soluble monomeric entity by GBM and breast cancer cells. This soluble ephrin A1 could function in a paracrine manner, induce EphA2 internalization and downregulation, elicit substantial alterations of cell morphology, and inhibit cell migration in treated GBM cells, in a juxtacrine interaction-independent manner [50]. Treatment with ephrin A1-conditioned media abolishes the phosphorylation of ERK induced by empty vector-conditioned media, which might contain growth factors. Moreover, treatment with a fusion protein of monomeric ephrin A1 (mEA1) also induced phosphorylation and degradation of in human breast cancer cells [51]. Thus, ephrin A1-associated tumor suppression might result from EphA2 downregulation as well as direct signaling through EphA2.

In addition to soluble ephrin A1, ephrin A1-Fc, obtained by fusing recombinant Ephrin A1 to human IgG Fc for dimerization, shows ephrin-like features and induces EphA2 phosphorylation [52]. Treatment with ephrin A1-Fc resulted in reduced amounts of membrane-associated EphA2 and inhibited cellular motility and invasion in pancreatic ductal adenocarcinoma cells [52]. In addition, proteasomal degradation was demonstrated to play a critical role in ephrin A1-Fc-associated EphA2 catabolism as the proteasome suppressor MG132 markedly inhibits ephrin A1-Fc-related EphA2 degradation. Likewise, ephrin A1-Fc increases EphA2 phosphorylation, decreases EphA2 protein expression, and inhibits growth in gastric cancer cells [53]. Dimeric ephrin A1-Fc suppresses Ras-mitogen-activated protein kinase (MAPK) signaling to reduce growth factor-associated ERK phosphorylation [92–95].

#### EphA2 monoclonal antibody

The large extracellular domain of EphA2 provides an antigen that is frequently upregulated on tumor cells [10, 96]. In addition, ligand stimulation is sufficient to induce EphA2 degradation. These evidences suggest that EphA2 could elicit a particularly attractive monoclonal antibody, and antibodies that mimic the actions of ephrin A1 would be expected to function similarly as the ligand.

Studies have shown that several agonist monoclonal antibodies raised against EphA2 induce its internalization and degradation, suppressing its malignant features. For example, Kinch et al. [45] isolated antibodies from mice after immunization with the pcDNA3-ecdEphA2-Fc expression plasmid, and identified EA1.2 that dose-dependently elevated phosphotyrosine amounts in EphA2. These authors demonstrated that EA1.2 inhibits more than 60% of soft agar-formed colonies in breast cancer cells compared with vehicle-treated controls. These findings indicate that the growth-suppressive effects of EphA2-specific antibodies correlate with their capability of stimulating EphA2 autophosphorylation and degradation. Coffman et al. [54] demonstrated that two antibodies, including EA2 and B233, promote EphA2 phosphorylation and degradation in cancer cells. The antibody EA2 (6 mg/kg) administered i.p. was shown to significantly decrease breast and lung cancer cell growth in vivo relative to the matched isotype controls (IgG1, 1A7). Goldgur et al. [55] isolated and characterized the anti-EphA2 single-chain antibody D2 scFv, which was highly specific to EphA2 and blocked ligand interaction in COS-7 cells. Indeed, treatment with D2 scFv induced apoptosis and reduced cell proliferation in the lymphoma cell line. In addition, Sakamoto et al. [43] showed that one of the EphA2 mAbs produced, SHM16, interacts with an EphA2 epitope differing from that affecting ephrin A1 binding to EphA2. SHM16 was clearly internalized in cells and inhibited malignant features in melanoma cells. However, SHM16 showed no effects on ephrin A1 interaction with EphA2 on the cell surface, while recognizing a different EphA2 epitope. SHM16 was shown to be clearly internalized by A375 cells.

Antibody-dependent cellular cytotoxicity (ADCC) kills cells via perforin/granzyme, TRAIL, and FasL [97]. ADCC also affects adaptive tumor immunity, and its enhancement could remarkably alter the tumor microenvironment [98, 99]. DS-8895a, a newly developed humanized anti-EphA2 mAb afucosylated for ADCC enhancement, was generated by mouse immunization with recombinant human EphA2 and further humanized as human IgG1 [56]. Treatment with DS-8895a of EphA2-positive breast and gastric cancer cells was shown to partially inhibit ephrin A1-associated EphA2 phosphorylation. In agreement, treatment with DS-8895a inhibits tumor growth in EphA2-positive human breast and gastric cancer xenografts in mice [57]. Another EphA2 effector-enhanced agonist monoclonal antibody that exhibits ADCC activity is 3F2-3M [44]. 3F2-3M was obtained by fusing the mouse parental antibody B233 and the humanized antibody 3F2. 3F2-3M administration dose-dependently increased EphA2 phosphorylation in the breast cancer cell line, which was similar to that of the parental antibodies 3F2-WT and B233 [44]. 3F2-3M significantly inhibited ovarian, breast, and lung cancer cell lines, which were co-cultured with peripheral blood monocytes from a healthy donor. However, 3F2-3M was minimally toxic in the absence of NK cells. On the other hand, interaction with NKs was increased by 100–250-fold for 3F2-3M in comparison with 3F2-WT, with improved affinity to FcγRIIIa. Modifying the Fc portion of the EphA2 antibody resulted in enhanced interaction with FcγRIIIa. Administration of 3F2-3M significantly induced tumor growth inhibition in a breast cancer xenograft orthotopic model, compared with the isotype control antibody and phosphate buffer saline (PBS) groups.

### Blocking EphA2 activation

Compounds binding to EphA2 or ephrin A1 could suppress signaling by direct antagonist effects.

#### Inhibiting Eph-ephrin interactions

Small molecules that block EphA2 could represent efficient alternatives to peptides and antibodies. Recently, small molecules disrupting the Eph-ephrin complex have been described, with most exerting pharmacological activities through targeting of the ligand-binding domain of EphA2, thereby acting as common protein-protein interaction (PPI) inhibitors. The ephrin binding site in Eph receptors allow high-affinity binding of small molecules [59, 100].

It was hypothesized that soluble receptors repress EphA signaling by suppressing the interactions of endogenous ephrins with EphA receptors. EphA2-Fc represents a soluble protein chimera involving the fusion of EphA2’s extracellular domain with human IgG1 Fc, preventing interactions of several ephrin A ligands with endogenous receptors and potently inhibiting EphA receptor activation in cultured cells. By interacting with ephrin A1, EphA2-Fc could induce ephrin-initiated reverse signaling. Treatment with EphA2-Fc was shown to dose-dependently inhibit EphA2 receptor phosphorylation and activity. In addition, EphA2-Fc strongly inhibited angiogenesis and microvessel growth in vitro as well as growth in pancreatic tumor xenografts [58]. Furthermore, soluble EphA2-Fc was demonstrated to inhibit endothelial cell migration upon 4 T1 mouse mammary adenocarcinoma tumor cell-induced angiogenesis in vitro. Moreover, EphA2-Fc inhibited 4 T1 tumor growth in vivo and reduced tumor vascular density and growth while increasing cell apoptosis.

Lithocholic acid (LCA, (3a,5b)-3-hydroxycholan-24-oic acid), a secondary bile acid produced by prokaryotic transformation of chenodeoxycholic acid, is considered an EphA2 antagonist. LCA interacts with the nuclear receptor farnesoid X receptor (FXR) and the G-protein-coupled receptor G-protein-coupled bile acid receptor 1 (GPBAR1, also called TGR5) under physiological conditions [101, 102]. Molecular modeling investigations revealed that LCA mimics ephrin A1 in interacting with EphA2 via insertion of its cyclopenta[a]-perhydrophenanthrene scaffold into the hydrophobic EphA2 receptor ligand-binding channel, generating a salt bridge involving Arg103 [60], an essential amino acid in ephrin A1 recognition [32]. LCA was shown to competitively and reversibly inhibit EphA2-ephrin A1 binding (Ki = 49 μM) without reducing EphA2’s kinase activity [59]. Further functional assays revealed that LCA inhibits EphA2 autophosphorylation and blocks ephrin A1-related prostate cancer cell cytotoxicity.

The specificity of LAC in antagonizing Eph receptor has been demonstrated, with no detected effects on other RTKs, including EGFR, vascular endothelial growth factor receptor (VEGFR), insulin-like growth factor 1 receptor (IGF-1R), and the insulin receptor. However, LCA is also considered to interact with EphA and EphB receptors, indicating an interaction with the highly conserved region of Eph receptor family members [59]. Thus, LCA has been used as a prototype for designing or identifying other PPIs. Amino acid conjugates of LCA were shown to effectively disrupt EphA2 binding to ephrin A1 and to suppress EphA2 phosphorylation in intact cells, thereby blunting malignancy. UniPR126 (N-(3a-hydroxy-5b-cholan-24-oyl)-L-tryptophan), a novel antagonist derived from LCA, inhibits EphA2 phosphorylation and angiogenesis in cultured cells, in the low micromolar range [61]. UniPR126 was shown to disrupt the EphA2-ephrin A1 complex and to inhibit EphA2 phosphorylation in prostate cancer cells at a level 6-fold higher (pIC50 = 4.89). UniPR129 (N-(3a-hydroxy-5b-cholan-24-oyl)-Lb-homotryptophan, the L-homo-Trp conjugate of LCA, another newly developed PPI based on the in silico model of the EphA2-UniPR126 complex, also disrupts EphA2-ephrin A1 interaction (IC50 = 945 nM; Ki = 370 nM) [62]. In agreement, UniPR129 was shown to inhibit ephrin A1-Fc-associated prostate cancer cell cytotoxicity and angiogenesis in vitro. In addition, both UniPR129 and UniPR126 reduce polygon formation, but UniPR129 (IC50 = 5.2 μM) was 4-fold more potent than UniPR126 (IC50 = 20.5 μM). IC50 values in inhibiting ephrin A1-related EphA2 phosphorylation were 5 and 12 μM, respectively, for UniPR129 and UniPR126. Furthermore, UniPR126 showed cytotoxicity in HUVECs, increasing lactic dehydrogenase (LDH) release, unlike UniPR129. Comparing efficacy for prostate cancer cell retraction, UniPR129 and UniPR126 had similar strengths, and were much more potent compared with LCA. Likewise, a series of L-Trp derivatives of LCA have been synthesized, and a compound (defined as compound 20) was identified as the most potent antagonist disrupting EphA2 binding to ephrin A1 [60]. This compound blocking EphA2 phosphorylation (IC50 = 12 μM) was 4–5 times more efficient compared with LCA (IC50 = 50 μM) in inhibiting prostate cancer cells. Treatment with compound 20 significantly reduced the percentage of retracted cells stimulated by ephrin A1-Fc. In addition, UniPR1331 (N-(3b-hydroxy-D5-cholen-24-oyl)-L tryptophan) was identified as the first orally bioavailable small molecule antagonizing the Eph-ephrin system [103]. UniPR1331 was obtained by conjugating L-tryptophan with the parent compound 3β-hydroxy-D5-cholenic acid, which serves as bioisostere analogues of LCA. The activity of UniPR1331 in blunting EphA2 binding to ephrin A1 (pIC50 = 5.45) was ten times increased compared with that of the parent 3β-hydroxy-D5-cholenic acid (pIC50 = 4.40), and barely stronger than LCA (pIC50 = 4.25). Administration of UniPR1331 was shown to inhibit GBM growth and to extend the time to progression in a subcutaneous xenograft model through inhibition of angiogenesis [63, 104]. Cholanic acid ((5b)-cholan-24-oic acid) is another molecule competitively inhibiting EphA2 binding to ephrin A1 with increased potency compared with LCA [64]. Cholanic acid has a specific and reversible interaction with EphA2’s ligand-binding domain, blocking EphA2 phosphorylation and prostate cancer cell cytotoxicity. In contrast to LCA (promiscuous binding), cholanic acid is more selective for EphA receptors. Cholanic acid inhibits Eph receptor phosphorylation at non-cytotoxic levels. It inhibits EphA2 activation by ephrins (IC50 = 12 μM) more effectively compared with LCA (IC50 = 46 μM) [64]. In addition, cholanic acid suppresses EphA2 phosphorylation via direct binding to the EphA2 kinase domain rather than inhibiting EphA2 kinase activity.

Besides LCA and its analogues, small molecules that interfere with the EphA2-ephrin A1 system comprise the following: (i) the FXR agonist GW4064 [65], a stilbene carboxylic acid, dose-dependently disrupts the EphA2-ephrin A1 complex (IC50 = 23 μM), inhibits EphA2 phosphorylation (IC50 = 31 μM) and blocks EphA2 activation in prostate cancer cells; (ii) the disalicylic acid-furanyl derivative 76D10 (5,5′-(5,5′-((1E,4E)-3-oxopenta-1,4-diene-1,5-diyl)bis(furan-5,2-diyl))bis(2-hydroxybenzoic acid) inhibits ephrin interaction with EphA2, reducing EphA2 phosphorylation stimulated by ephrin-A1 Fc and inhibiting EphA2-mediated cell retraction in prostate cancer cells [66].

#### Inhibiting kinase activity of EphA2

The successful development of specific RTK inhibitors has prompted subsequent efforts for identifying comparable targets. Unlike other anticancer approaches, targeted therapies are relatively less toxic. Multiple small molecule EphA2 inhibitors interacting with the intracellular kinase domain have been described.

Dasatinib (BMS-354825) represents an oral kinase inhibitor simultaneously targeting breakpoint cluster region-Abelson (BCR-ABL), c-KIT, platelet-derived growth factor receptor (PDGFR), and SFKs [105, 106]. Its anticancer features have been demonstrated in early- and late-phase clinical studies of chronic myelogenous leukemia (CML). A variety of studies have demonstrated that dasatinib directly reduces EphA2 phosphorylation and kinase activity [67, 68, 107]. However, the promiscuous targeting profile of dasatinib makes data interpretation ambiguous. Dasatinib has also been recently used as a lead structure for developing EPHA2-inhibitors with ameliorated targeting profiles. The novel EphA2 inhibitor candidate 4a based on dasatinib was shown to feature an ameliorated selectivity profile while maintaining potent inhibitory effects against EphA2 as well as cytotoxic properties in glioblastoma cells [69].

ALW-II-41-27, a type II small molecule inhibitor targeting the ATP-binding region of the kinase domain as well as an allosteric site following the “DFG” motif in EphA2, has been shown to bind to and potently inhibit EphA2 kinase activity [70, 108]. Treatment with ALW-II-41-27 inhibits EphA2 kinase activity in NSCLC cells (IC50 = 11 nM) and suppresses cell survival and proliferation, while inducing cell apoptosis in vitro [70]. In the NSCLC xenograft model, oral treatment with ALW-II-41-27 revealed a relatively poor pharmacokinetic profile and low oral bioavailability. Mice treated with ALW-II-41-27 intraperitoneally showed significantly inhibited tumor growth. In addition, an in vivo study confirmed ALW-II-41-27 specificity for EphA2 among Eph receptors, although significant interactions were detected with multiple intracellular kinases such as Abelson (ABL), p38 MAPK, and many steroid receptor coactivator (SRC)-family kinases. ALW-II-41-27 was also shown to decrease both survival and proliferation in cultured erlotinib-resistant lung cancer cells, inhibiting tumor growth in mouse xenografts [25]. Furthermore, no statistical difference in body weight was detected, and no significant histopathologic differences were found in the heart, liver, or kidney tissue in mice treated with ALW-II-41-27 [70].

### EphA2 as drug delivery target

Peptides and antibodies selectively binding cancer cells followed by internalization provide a powerful vehicle to guide therapeutic delivery to specific cell types and to determine the format of peptide/antibody-drug conjugates or antibody-directed nanotherapeutics. These conjugates or targeted systems could deliver toxic compounds selectively to the tumors while sparing noncancerous tissues. For example, EphA2 could selectively deliver therapeutics to EphA2-overexpressing cancers while simultaneously regulating EphA2-signaling related events.

#### Peptide/antibody-drug conjugates

Ephrin A1-Fc has been utilized in EphA2 targeted therapy upon conjugation with PE38QQR, a derivative of Pseudomonas aeruginosa endotoxin A, to generate ephrin A1–PE38QQR conjugated cytotoxin [71]. Pseudomonas endotoxin A is a bacterial toxin with high cytotoxicity in eukaryotic cells; it can be genetically modified by replacing the natural eukaryotic cell receptor binding domain by a tumor-specific target antibody or ligand [109, 110]. It was shown that EphA2 protein levels are significantly decreased following ephrin A1–PE38QQR administration in glioblastoma cells [71]. Unsurprisingly, ephrin A1–PE38QQR was cytotoxic to glioblastoma, breast cancer, and prostate cancer cells that overexpress EphA2.

1C1 is a fully human monoclonal antibody with selective binding to EphA2, but no other Eph receptor family member [72]. After cell binding in prostate cancer cells, 1C1 rapidly induces tyrosine phosphorylation, internalization, and degradation of EphA2. However, 1C1 does not demonstrate direct cytotoxicity and antitumor effects, but allows highly toxic chemotherapeutics to be directly and specifically delivered to EphA2-expressing tumor cells. The EphA2 immunoconjugate MEDI-547 (1C1-mcMMAF) was generated by conjugating 1C1 with the chemotherapeutic drug monomethyl auristatin phenylalanine (MMAF) via the non-cleavable linker maleimidocaproyl (mc) [72]. MEDI-547 was shown to interact with EphA2 via the highly conserved extracellular domain with similar binding affinity observed for 1C1, with internalization in EphA2-expressing tumor cells and subsequent reduction of EphA2 protein levels. In vitro experiments revealed that MEDI-547 decreases viability and increases apoptosis in ovarian, endometrial carcinoma cells in an EphA2-specific fashion [73, 74]. Indeed, administration of MEDI-547 significantly reduced tumor growth with minimal adverse effects in mice and rats (evaluated by body weight lose). In addition, mice treated with MEDI-547 showed decreased rate of distant metastasis.

#### Antibody-directed nanotherapeutics

Off-target drug toxicity frequently causes treatment discontinuation, restricted dose escalation, and worse outcomes. Antibody-mediated tumor targeting and nanoparticle encapsulation decrease the toxic effects of anticancer agents, improving treatment efficacy. Trametinib (TMB) is a MAP/ERK kinase (MEK) inhibitor, but its off-target toxicities frequently prompt dose interruption as well as treatment discontinuation [111]. Thus, YTPL, an ephrin A1-mimicking peptide (YSA; amino acid sequence: YSAYPDSVPMMS) with high stability [112], has been anchored on TMB-loaded PEGylated nanoliposomes [75]. YTPL was shown to display elevated cell internalization in comparison with non-targeted nanoliposomes (TPL) due to receptor-associated uptake. Due to elevated EphA2 amounts in vemurafenib-resistant cells in comparison with parent cells, YTPL shows higher intracellular uptake in the former cells. In addition, TMB was confirmed to be released upon TPL internalization in tumor cells. Such a delivery approach markedly reduces the amounts of circulating free TMB, consequently minimizing undesirable effects. Likewise, MM-310 (EphA2-ILs-DTXp) are immunoliposomes encapsulating the readily hydrolysable docetaxel prodrug (DTXp) with conjugation to the high-affinity signal-chain variable fragment (scFv-3) targeting EphA2 [76, 113]. Administration of MM-310 was shown to remarkably enhance anticancer activity in multiple mouse tumor xenografts (from breast, prostate, gastric, and esophageal cancer cells), in comparison with the free docetaxel and non-targeted nanotherapeutic control groups [76]. Moreover, pharmacokinetic analysis revealed that the AUC of docetaxel was increased by 15-fold. Delivery via MM-310 resulted in slow and sustained release of DTXp, decreased circulatory amounts of active docetaxel, and significantly reduced hematologic toxicity in comparison with docetaxel. Administration of MM-310 maintained adequate drug levels in tumors. These findings suggest that MM-310 has improved pharmacokinetic features, with reduced plasma docetaxel and selective tumor exposure, resulting in ameliorated toxicity profile and augmented anticancer effects. Based on these findings, a phase 1 clinical trial was initiated for evaluating the effectiveness of MM-310 in many solid tumors (ClinicalTrials.gov: NCT03076372).

### EphA2-based immunotherapy

Immunotherapy, which relies on enhancing the patient’s immune defenses to combat tumor cells, has become a game changer in cancer treatment. EphA2 overexpression on tumor cells could constitute a new antigen for tumor immunotherapy.

#### Vaccines

Dendritic cell (DC)-based vaccines represent attractive anticancer tools, since DCs induce both tumor antigen-specific cytotoxic T lymphocytes (CTLs) and helper T cells [114]. Tumor antigen-derived peptide-DC vaccines could result in improved clinical outcome. Yamaguchi and collaborators [77] evaluated immunization with DCs pulsed with EphA2-derived peptides (Eph-DCs) in a mouse model of colorectal cancer, demonstrating the inhibition of relevant EphA2-positive mouse colon carcinoma MC38 cell-subcutaneous xenografts in comparison with the unpulsed DC and PBS groups. Interestingly, there was no significant difference in EphA2-negative melanoma BL6 cell-derived subcutaneous xenografts between the Eph-DC and unpulsed DC groups, suggesting vaccination with Eph-DCs provides specific antitumor effects against tumors positively express EphA2. In addition, both CD4+ and CD8+ CTLs, but not natural killer (NK) cells, were required for the anticancer effects detected upon immunization with Eph-DCs. The authors further demonstrated that treatment with Eph-DCs in mice results in higher tumor-specific CTL activity, in comparison with unpulsed DCs [78]. The MC38 and BL6 cell-derived intrahepatical xenografts in mice immunized with both Eph-DCs and unpulsed DCs were markedly inhibited in comparison with the PBS treatment group. Finally, Eph-DC immunizations were more effective against rechallenged tumor, consistent with their superior capacity to elicite EphA2-specific CTLs.

#### CAR-T

A strategy to treat cancer is chimeric antigen receptors (CARs) modified T (CAR-T) cell therapy, a cell-based tumor immunotherapeutic approach [115, 116]. CAR-T cells are T cells genetically engineered for producing a tumor targeting receptor, with normal T cells modified to recognize specific antigens for tumor cell targeting. The receptor combines a signaling domain of the T cell receptor (TcR) complex and an antigen-binding domain, e.g., an antibody’s scFv [117]. Therefore, independently of the native TcR, CAR-T cells recognize cancer cells through the CAR receptor. CAR-T cells that target CD19 show stark anticancer effects for chemo-refractory B cell-derived hematological cancers, which has resulted in FDA approval [118].

Several EphA2-specific T cells have been developed and evaluated in preclinical studies, recognizing EphA2-expressing tumors as assessed by interferon-γ (IFN-γ) and IL-2 synthesis and conferring cancer cell cytotoxicity. A second-generation EphA2-specific CAR was engineered on the basis of the humanized EphA2 monoclonal antibody 4H5, a CD28.ζ signaling domain, a CD28 transmembrane domain, and a CH2CH3 spacer. The final T cell populations comprised both CD4+ and CD8+ cells, all expressing EphA2-specific CARs [79]. These EphA2-specific T cells were demonstrated to identify and kill glioblastoma cells expressing EphA2. In addition, treatment with these EphA2-specific T cells suppressed EphA2-positive U373 glioma xenografts in severe combined immunodeficiency (SCID) mice and markedly increased animal survival in comparison with non-treated mice and those administered non-transduced T cells [79]. However, the CH2CH3 spacer might compromise the anticancer effects of CAR-T cells in vivo by promoting T cell sensitivity to immune cells expressing Fc receptors [119]. The EphA2-specific T cells were subsequently improved via CH2CH3 spacer replacement with an IgG1-derived short spacer, increasing the anti-glioma effects of CD28.ζ CAR T cells by 20-fold [80]. In addition to targeting gliomas, EphA2-specifc T cells have also been developed and evaluated in NSCLC [81] and esophageal squamous cell carcinoma (ESCC) [82]. Of interest, these tested EphA2-specifc T cells were shown to exhibit the ability to kill EphA2-positive tumor cells [81, 82]. In addition, administration of EphA2-specifc T cells results in inhibited lung cancer in vivo [81]. However, mice administered both EphA2-specifc and non-transduced T cells died within 7–8 weeks from non-tumor causes, which deserves further investigation.

### EphA2-based combination therapeutics

Various modalities of combination therapy based on EphA2 targeting have been evaluated in preclinical studies (Table 2). For example, combined use of ALW-II-41-27 and WW437, a histone deacetylase inhibitor that could suppress phosphorylated EphA2 and EphA2 expression, results in remarkably increased effects on breast cancer cell growth and migration compared with either drug administered as monotherapy [87]. In another study, Martini et al. [27] demonstrated in cetuximab-resistant colorectal cancer cells a more pronounced EphA2 activation in comparison with sensitive ones. Joint administration of ALW-II-41-27 and cetuximab was shown to revert primary and acquired resistance to cetuximab, inhibit proliferation, and induce apoptosis in cultured cells. Likewise, significantly decreased growth of xenografts in vivo was found compared with the cetuximab alone group. Zhou et al. [86] showed in glioma cells that treatment with siRNA EphA2 exerts almost the same cell growth inhibitory effects as 3 chemotherapeutics, including cisplatin, etoposide, and minustine hydrochloride. Combining siRNA EphA2 and these anticancer agents markedly enhanced their effects. In addition, UniPR1331 was also shown to significantly increase the efficacy of bevacizumab, further reducing tumor growth in glioblastoma cells in in vivo mouse xenografts [63]. Also of interest, a study by Hasegawa et al. showed cisplatin alone does not suppress SNU-16 tumor growth at 10 mg/kg; however, combination with DS-8895a resulted in a therapeutic benefit in comparison with administration of the drug alone [56]. In a mouse breast cancer model, combination of MM-310 with anti-PD-1 (anti-mouse PD-1 antibody J43 and anti-PD-L1 antibody MPL3280) resulted in a 60% complete response rate, with durable responses that were resistant to re-challenge [76]. This combination resulted in a 93% TGI, which was greater than the effect observed with MM-310 and anti-PD-1 as monotherapies (81% and 54% TGI, respectively).

**Table 2:** Combination therapeutics based on EPHA2 targeting

Drug	Cancer type	Effects in vitro	Effects in vivo	Ref.
ALW-II-41-27 + WW437	Breast cancer	Inhibit cell viability and migration	-	[87]
ALW-II-41-27 + cetuximab	CRC	Inhibit cell growth, induce apoptosis and cell cycle arrest, revert resistance to cetuximab	Inhibit tumor growth	[27]
siRNA+ cisplatin/etoposide/minustine hydrochloride	Glioma	Induce cytotoxicity	-	[86]
UniPR1331 + bevacizumab	Glioblastoma	–	Inhibit tumor growth	[63]
DS-8895a + cisplatin	Gastric cancer	–	Inhibit tumor growth	[56]
EphA2-ILs-DTXp + PD-1 inhibitors	Breast cancer (mouse)	–	Inhibit tumor growth	[76]

## EphA2-based clinical development

Based on the preclinical studies mentioned above, several therapies have entered clinical trials, including dasatinib, MEDI-547, DS-8895a, BT5528, MM-310, EphA2-targeting DOPC-encapsulated siRNA, vaccine, and CAR-T cell immunotherapy. Dasatinib represents the only molecule already administered to humans in multiple clinical studies of cancer. However, dasatinib is frequently used as a BCR-ABL kinase inhibitor for treating malignant diseases. EphA2 is used as a biomarker for assessing patient response to dasatinib. Unfortunately, few of the remaining EphA2-target therapies have exhibited successful clinical outcomes. Drugs targeting EphA2 in clinical trials are shown in Table 3.

**Table 3:** Key clinical trials of EphA2 targeted therapy in cancer

Agent or approaches	Trial identifier	Study characteristics	Intervention	Status
MEDI-547	NCT00796055	Phase 1, *n* = 6 EphA2-positive solid tumor	IV infusion with MEDI-547 0.08 mg/kg on day 1 of 21-day cycle Primary outcome: safety and tolerability	Terminated
DS-8895a	NCT02252211	Phase 1, *n* = 9 EphA2-positive solid tumor	Infusion with ^89Zr-Df-DS-8895a 0.2 mg/kg i.v on day 1. DS-8895a 1, 3, or 10 mg/kg on days 8 and 22, and ^89Zr-Df-DS-8895a 1, 3, or 10 mg/kg on day 36 Primary outcome: toxicity	Completed
DS-8895a	NCT02004717	Phase 1, *n* = 37 Solid tumor	Step 1: IV infusion with DS-8895a 0.1, 0.3, 1, 5, 10, or 20 mg/kg on day 1 of 14-day cycle; step 2: IV infusion with DS-8895a 20 mg/kg on day 1 of 14-day cycle Primary outcome: toxicity, serum pharmacokinetics	Completed
CAR-T cell	NCT02575261	Phase 1/2, *n* = 60 EphA2-positive glioma	Primary outcome: effectiveness	Completed
CAR-T cell	NCT03423992	Phase 1, *n* = 100 Recurrent glioma	Primary outcome: adverse events	Ongoing
BT5528	NCT04180371	Phase 1/2, *n* = 152 Solid tumor	IV infusion of BT5528 once a week (days 1, 8, 15, and 22) on a 4-week cycle with or without 480 mg nivolumab Primary outcome: safety, MDT	Ongoing
SiRNA-EphA2-DOPC	NCT01591356	Phase 1, *n* = 40 Advanced solid tumor	IV infusion of siRNA-EphA2-DOPC on days 1 and 4 of 21-day cycle Primary outcome: safety, MDT, and ORR	Ongoing
MM-310	NCT03076372	Phase 1, *n* = 34 Solid tumor	IV infusion of MM-310 on day 1 of 21-day cycle Primary outcome: MDT	Ongoing
Vaccine	NCT02754362	Phase 2, *n* = 30 Recurrent glioblastoma	Block 1: bevacizumab every 2 weeks for 2 doses; block 2: vaccine + poly-ICLC + bevacizumab on weeks 1, 3, 5, and 7; block 3: vaccine + poly-ICLC monthly and bevacizumab every 2 weeks for 10 months Primary outcome: immune response, tumor response	Active, not recruiting

The abovementioned preclinical antitumor effects of DS-8895a on EphA2-overexpressing tumor cells advocate for its further clinical development. There are currently two phase I open-label studies determining the safety, tolerability, and pharmacokinetic features of DS-8895a in individuals with advanced-stage solid tumors (NCT02004717, NCT02252211). The NCT02004717 trial was a two-step, study with step 1 evaluating a dose escalation cohort (six dose levels from 0.1 to 20 mg/kg) in patients with advanced solid tumors and step 2 assessing dose expansion in individuals with EphA2-positive esophageal and gastric cancers. The maximum tolerated dose was not reached in step 1 and the planned highest dose (20 mg/kg) was used in step 2. A total of 37 cases (22 and 15 in steps 1 and 2, respectively) were included, but all discontinued the study for overt disease progression (20 and 13 in steps 1 and 2, respectively) or adverse events (AEs, the remaining cases) [120]. Similarly, the NCT02004717 trial enrolled 9 patients, who did not complete the study. There were 55.56% patients (5/9) with progressive disease and 22.22% (2/9) with serious adverse events, including cancer pain and spinal cord compression.

The safety, pharmacokinetic features, and anticancer effects of MEDI-547 were evaluated in a phase 1, open-label trial with included dose-escalation and dose-expansion cohorts [121]. Cases underwent a 1-h intravenous infusion of MEDI-547 (0.08 mg/kg) at 3-week intervals. This trial enrolled 6 patients but all discontinued the therapy due to treatment-associated bleeding (*n* = 3) and coagulation (*n* = 2) events, and the planned dose escalation was not pursued. Clinical responses comprised disease progression (*n* = 5, 83.3%) and stable disease (*n* = 1, 16.7%). The safety profile of MEDI-547 prevents its further clinical development for advanced-stage solid tumors. However, the causes of the detected AEs remain unclear.

## Perspective and conclusion

The crucial roles in tumor biology have defined EphA2 as a promising therapeutic target. Due to intensive investigation and remarkable advances in understanding some of the mechanisms associated with EphA2 effects, multiple potential targets have been described. The advantages of the EphA2 as the target of tumor therapy include (i) possibility of targetable by cellular, molecular, and pharmaceutical approaches; (ii) possibility of developing anticancer immune-therapy; and (iii) possibility of combination with conventional therapeutics to improve efficacy.

However, to date, the precise mechanisms of EphA2, especially the effects of EphA2:ephrin reverse signaling, are largely unknown, and the outcomes of its regulation cannot be predicted with confidence. Indeed, EphA2-ephrin A1 signaling is extremely complex, with both interacting cells receiving interdependent signals from the identical signaling complex that is frequently associated with other Eph receptors and receptor tyrosine kinases, as well as additional signaling pathways. Meanwhile, EphA2 responses following stimulation could result from cell/tissue-specific kinase-dependent or kinase-independent signaling pathways. However, most of the preclinical work presented in this review tend to simplify the EphA2-ephrin A1 system. EphA2 inhibition or targeting confers marked benefits in experimental studies. However, these findings could not be translated into clinical use. For the currently developed EphA2-based targets, existing challenges include the following: (i) downregulation of EphA2 by siRNA affects both the forward and reverse signaling, together with compensatory stimulation of other Eph receptors and oncogenic signalings, which potentially regulate the biological behavior of a cell; (ii) compound that act on the extracellular ligand-binding domain of EphA2 block both reverse and forward signaling, and show poor physicochemical characteristics; (iii) the highly conserved cytoplasmic domain, particularly the kinase domain, among different Eph kinases, could lead to non-specific inhibition of other Eph family members and unwanted toxicities by the use of small molecule inhibitors to antagonize EphA2’s enzymatic activity; (iv) although antibody-drug conjugates are highly specific and stable, and possess antibody-like pharmacokinetic features, the non-negligible technological limitations impact drug activity and/or safety; (v) the significant proportion of non-responsive cases and treatment-related toxicities remain obstacles to successful immunotherapy treatment.

Sustained and systematic efforts in the future may involve an in-depth understanding of EphA2-ephrin A1 signaling and a precise elucidation of the crosstalk with other oncogenic pathways. The performed clinical studies and novel biological findings provide clues for developing next-generation EphA2-targeting therapies: (i) focus should be placed on enhancing efficacy and selectivity while preventing off-target secondary effects; (ii) combination with other therapies may be helpful. Despite multiple challenges due to the complex biological properties of the EphA2-ephrin A1 system, exciting possibilities still exist for novel treatment approaches based on these molecules.

## Data Availability

Not applicable.

## Abbreviations

ADCC: Antibody-dependent cellular cytotoxicity
AEs: Adverse events
AONs: Antisense oligonucleotides
BCR-ABL: Breakpoint cluster region-Abelson
CARs: Chimeric antigen receptors
CAR-T: (CARs) modified T cell
CML: Chronic myelogenous leukemia
CTLs: Cytotoxic T lymphocytes
DC: Dendritic cell
DFS: Disease-free survival
DOPC: 1,2-dioleoyl-sn-glycero-3-phosphatidylcholine
DTXp: Docetaxel prodrug
ECM: Extracellular matrix
eck: Epithelial cell kinase
EGF: Epidermal growth factor
EGFR: Epidermal growth factor receptor
Ephrin: Eph receptor-interacting
ER: Estrogen receptor
ERK: Extracellular regulated protein kinases
ESCC: Esophageal squamous cell carcinoma
FAK: Focal adhesion kinase
FcγRs: Fcγ receptors
FXR: Farnesoid X receptor
GBM: Glioblastoma multiforme
GPBAR1: G protein-coupled bile acid receptor 1
HUVECs: Human umbilical vein endothelial cells
IFN-γ: Interferon-γ
IGF-1R: Insulin-like growth factor I receptor
IgG: Immunoglobulin g
LCA: Lithocholic acid
LDH: Lactic dehydrogenase
LMW-PTP: Low-molecular-weight protein phosphatase
MMAF: Monomethyl auristatin phenylalanine
MEK: MAP/ERK kinase
NK: Natural killer
NSCLC: Non-small cell lung cancer
MAPK: Mitogen-activated protein kinase
mEA1: Monomeric ephrin A1
OS: Overall survival
PBS: Phosphate buffer saline
PDGFR: Platelet-derived growth factor receptor
Raf: Rapidly accelerated fibrosarcoma
Ras: Rat sarcoma
PPI: Protein-protein interaction
RNAi: RNA interference
RTKs: Receptor tyrosine kinases
RTKs: Receptor tyrosine kinases
SCID: Severe combined immunodeficiency
sgRNAs: Single guide RNAs
siRNAs: Short interfering RNAs
SRC: Steroid receptor coactivator
TcR: T cell receptor
TKIs: Tyrosine kinase inhibitors
TMB: Trametinib
UV: Ultravioletray
VEGFR: Vascular endothelial growth factor receptor
